# Positive and Healthy Living Program Manual Development for Young People Living With HIV at the Comprehensive Care Center at the Kenyatta National Hospital: An Open Pilot Implementation Trial

**DOI:** 10.3389/fpsyt.2020.487648

**Published:** 2020-11-17

**Authors:** Judy Machuka, Grace Nduku Wambua, Otsetswe Musindo, David Bukusi, Violet Okech, Peter Muiruri, Rachel Maina, Nelly Opiyo, Pauline Ng'ang'a, Manasi Kumar

**Affiliations:** ^1^CoEHM Project, Kenyatta National Hospital, Nairobi, Kenya; ^2^Department of Clinical, Neuro- & Developmental Psychology, Vrije University, Amsterdam, Netherlands; ^3^Kenyatta National Hospital, HPU/VCT/Youth Centre 2, Nairobi, Kenya; ^4^Department of Mental Health, Kenyatta National Hospital, Nairobi, Kenya; ^5^Department of Psychiatry, College of Health Sciences, University of Nairobi, Nairobi, Kenya; ^6^Research Department of Clinical London, UK Health & Educational Psychology, University College London, London, United Kingdom

**Keywords:** depression, adjustment, internal stigma, self-esteem, common elements therapeutic approach, HIV, young people

## Abstract

**Background:** Human Immunodeficiency Virus/Acquired Immune Deficiency Syndrome (HIV/AIDS) remains a great challenge among young people in Kenya. Young people living with HIV are faced with a lot of challenges that are often overlooked and may have an impact on their treatment adherence and overall well-being. This calls for interventions that are age-appropriate and which tap into the psychosocial problems they experience. This is a protocol of a proposed study aimed at developing a facilitator-led peer support manual called the “Positive and Healthy Living Program” that will be the basis for running support groups with young people at the Comprehensive Care Center (CCC) at the Kenyatta National Hospital (KNH).

**Methods:** We will carry out our study in two phases. The first phase will focus on the development of the manual and training of peer-facilitators. The second phase will make use of a pilot trial research design using both qualitative and quantitative approaches. It will be carried out among 10–24 year-olds attending CCC at KNH, and will consist of three groups: Tumaini Group (10–14 years), Amani Group (15–19 years), and Hodari Group (20–24 years). The groups will participate in an eight-session support group, whose activities will focus on four domains: social-recreation, psychotherapy, peer-modeling, and psychoeducation. Quantitative data will be collected using laboratory measures of Viral Load and CD4 as well as socio-psychological assessment tools. Qualitative data will be collected through interviews with the young people and peer facilitators. We will conduct a descriptive analysis which will describe the key features of the dataset and bivariate analyses will examine the association between variables. The change will be measured at baseline and post-treatment. The interviews will be coded into themes and we will generate experiential categories from the data around the effectiveness of the program, the peer facilitators' experience of providing support, how the young people respond to the program, and its influence on their overall well-being.

**Discussion:** We expect that the peer facilitators will find this manualized treatment acceptable and the eight-sessions group intervention will be feasible for the three age groups. We hypothesize that there will be improvements detected with regards to reported adherence and viral load, self-esteem, depression, and psychological functioning.

## Introduction

The World Health Organization's Global Accelerated Action for the Health of Adolescents (WHO-AA-HA) (1) has been promoting an age differentiated approach to treating adolescent health problems, given that new HIV infections and risks continue to make young people susceptible. The United Nations Program on HIV/AIDS (UNAIDS) (2) cited Kenya as the fourth largest center of the HIV/AIDS epidemic in the world (alongside Mozambique and Uganda), with 1.6 million people living with HIV. According to a Kenyan Ministry of Health/National AIDS Control Council (NACC) (3) report, more than half (51%) of all new HIV infections in Kenya in 2015 occurred among young people (aged 15–24 years). This is a rapid rise from 29% in 2013. An estimated 435,225 young people (ages 10 to 19) are HIV positive, while another 119,899 have the virus “but are not yet identified” or do not know their HIV status, suggesting “…*young people, especially young women, still bear the brunt of [the] HIV epidemic due to limited access to information, services, stigma, and discrimination*.” (4).

Young people who have had HIV/AIDS since birth face several challenges, such as difficult childhoods characterized by frequent hospitalization, poor school attendance, a small stature, delayed puberty, intellectual impairment, and skin disfiguration (5–8). Many have lost one or both parents to HIV/AIDS (9, 10). The psychosocial challenges include disclosure of their HIV status and potential stigma once disclosure has taken place. These are closely accompanied by challenges faced in daily living, access and adherence to treatment, access to care and support, a healthy sexual and social identity, gaining independence, coping and living positively with HIV/AIDS, their transition to adulthood, and clinic transition (11–13). Despite the overwhelming numbers seen in the Sub-Saharan African (SSA) context, there is still a gap in the treatment, care, and support programs that are specialized in addressing the needs of young people living with HIV (YLHIV) (14, 15).

The HIV/AIDS epidemic has severely impacted the mental health of young people (16, 17). A few studies suggest that this group may be at a greater risk for psychiatric problems or have poorer mental health compared with their uninfected peers (6, 18, 19). Mental health functioning is among the most significant predictors of health and behavioral outcomes. Thus, mental health problems may be a predisposing factor for HIV infection or a perpetuating factor for risky behavior in HIV-infected youth (20, 21). According to Vranda and Mothi (22), young people with chronic illness are at greater risk of psychiatric problems including depression, anxiety, and feelings of isolation. This is due to the complexity of their illness and treatment as well as adverse psychological circumstances and lives lived in distressed areas affected by poverty, violence, family conflict, substance use, and limited access to care (23).

There are few evidence-based mental health and health promotion programs supporting families to promote the health and psychosocial well-being of YLHIV in SSA (18). Despite the availability of antiretroviral treatments (ART), young people still experience a set of complex issues related to identification, care, and treatment (24–26). Young people are an ever-growing part of the HIV/AIDS epidemic. There is a gap in psycho-social support, life skills education, and peer support group interventions for this fast-growing HIV positive population in SSA generally and Kenya specifically. Therefore, there is a need for services geared toward HIV-positive young people that are age appropriate because they have specific needs that cannot be met through child or adult clinics (27). Without creating a separate space for youth-friendly services, this already vulnerable population could get lost in the current cascade of care into which they do not fit (28). Creating developmentally appropriate services for HIV-positive young people would help open vital communication links between them and healthcare personnel, which may help them stay engaged in care and thus facilitate health promoting behaviors (29). Contrastingly, age- appropriate transitional services for young people have been associated with improved follow-up, better disease outcomes, and improved psychological health.

This is a study protocol for a proposed study whose aim is to develop a peer-led manualized program called the “Positive and Healthy Living Program.” This program will be based on running support groups for young people at the CCC at KNH, with a focus on evidence-based, culturally and age appropriate activities. Finally, this study will explore whether the manual content is usable within the three groups of young people: *Tumaini* (meaning hope in Kiswahili, which comprises of participants aged 10–14 years), *Amani* (meaning peace, which comprises of participates aged 15–19 years), and *Hodari* (meaning courage, which comprises of participants aged 20–24 years). It will also assess acceptability (established through participants responses), feasibility (established through consistent participation, positive feedback of young people, improvements in treatment experience, and enhanced psychosocial support), fidelity (established through facilitators' reactions to content delivery), and short-term sustainability (established through integration into psychosocial programming at the CCC at KNH).

## Trial Conceptual Model

The WHO AA-HA (1) recommends the use of low intensity evidence-based interventions to address the unique needs of vulnerable young people. Our themes will be guided by a standardized adolescent package of care (APOC) for YLHIV developed and piloted by the Kenyan Ministry of Health (30). APOC focuses on stages of adolescent development, clinical assessments, mental health, communication and counseling, nutritional care and support, psychosocial support, sexual and reproductive health, and community level care and support, as well as adolescent/youth friendly services that support the transition of ALHIV from pediatric to adult care. In extending the APOC further we will adopt a common elements treatment approach (CETA): an eclectic framework that combines strategies from various psychotherapies. CETA is an approach which can be used to treat multiple common problems such as depression, anxiety, and other behavior problems faced by young people living with HIV. This approach makes use of evidenced-based elements from existing treatments and it is more effective than a single-focused treatment. The goal in using existing treatments is to develop a more simplified and flexible approach that involves skills that are easier to learn, simple language, and a short manual with practical guidelines. In adapting these therapies to a group of YLHIV, no substantial changes in the mainstay/core elements of therapies will be needed. Instead, adaptations and modifications to the settings, as well as peripheral issues such as terminologies, analogies, cultural factors, and contexts, will be made (31–33). For this proposed study we will use the APOC and CETA approach to develop a support group manual for this group. We will design an eight-session intervention that provides skills that are commonly used in evidence-based practices. We will achieve this by borrowing an implementation framework where these common therapeutic elements are collated, and adopters and mental health systems' ability to innovate these treatments are carefully studied.

We will conceptualize our common therapeutic elements in four tiers:

1. Social-recreational tier—to provide a social and recreational association with group work.
2. Peer modeling tier—to use peer support to moderate the session and a counselor or social worker to lend support. It also involves fostering peer mentorship and using peer feedback on problem solving as well as sharing/learning from personal stories of successes or failures in participants' own health management.
3. Psycho-educational tier—to provide greater support in terms of relevant, positive, and motivational, as well as HIV/AIDS disease related, information and support in measured doses so that the participants are not overwhelmed by the information, but use it to enhance their psychosocial functioning.
4. Psychotherapeutics tier—to assess and address specific needs such as motivational support, depression, anxiety, stigma, and shame. It puts a greater emphasis on understanding and tapping into emotional and thought disturbances, using strategies that young participants can adopt.

In this modular approach, we will try to elicit competencies that the participants and peer facilitators will develop through the support group program, therefore making the task of “CETA adaptation” and providing a structured therapy in the resource-constrained environment with high-risk patients more manageable. We will try to incorporate what Chorpita and Daleiden (34) call the move away from the “uptake of evidence-based strategies” toward the “generation of positive outcomes” for this vulnerable group of young people. The model of peer support and peer training is also an example of such a move from investing in specialist trainers to training lay counselors and peers (35) and we have looked at this issue further in Wambua et al. (36).

## Methods

### Phase 1: Manual Development

#### Data Collection Strategy

During the first phase, we will focus on developing the manual. We will begin with a needs assessment and analysis of psychosocial concerns and challenges facing young people. This information will be used in the development of age appropriate content and activities corresponding to four domains: socio-recreation, peer modeling, psychotherapy, and psychoeducation.

#### Manual Development Strategy

Content themes and activities will be developed further from psychosocial concerns identified in Phase 1 of the study (see Table 1). See Table 2 for an overview of the proposed session themes and activities. The activities will be expected to address the age—appropriate concerns for our three groups, leading to the development of separate manuals for each age group. We will maintain the same themes through each session in each group while tailoring the activities for each specific age group. The intervention will consist of six major themes, each to be delivered—in ~2 h—to mixed-gender groups facilitated by a male and a female peer-facilitator. The peer-facilitators will receive training to be able to relay the content. The modes of learning will vary from mini-lecturers, short videos, role plays, written activities in workbooks, discussions, and practice opportunities for skills building. Strategies that will be used include: bead making to build group cohesion; videos conveying key messages through culturally relevant examples; group discussions to explore key messages, generate ideas, and promote self-reflection; role-play with peers to build effective communication and negotiation skills; and workbook exercises to provide opportunities to note down new information and skills for use.

**Table 1:** Domain and parameters in data collection.

**Construct and domain**	**Measure and parameter**
**Phase 1: Month 1–3**	
1. Identifying psychosocial concerns and challenges facing adolescents	- CCC Records (case notes, viral load, treatment regimen and adherence practices)
	- Focus group discussion (FGD) with study team and CCC psychosocial team about the challenges and concerns of the adolescents to map out manual development
	- Sensitization about the study through ongoing support groups and recruitment of adolescent participants with collaborative effort from the study team and psychosocial team. Information about adolescent concerns obtained from support groups
2. Development of manual content and workbooks with activities corresponding to the four domains: socio-recreation, peer modeling, psychotherapy, psychoeducation	−4 domains and corresponding listed competencies/problem areas (see Table 2)
	- Manual content and workbooks developed in a consultative way with the study team and CCC Psychosocial team in FGDs using role plays
3. Training of facilitators and resource persons	−2 day training for 8 facilitators (Peer mentors from the CCC Psychosocial team)
	- Pre and post- training evaluation—testing the manual and workbook activities, awareness by participants, evaluation from participants and trainers, and *in vivo* observation
**Phase 2: Month 4–11**	
	- Baseline assessments (Sociodemographic questionnaire, Viral Load and CD4 lab test and psychological tools)
Intervention: open Trial to test the following domains	
1. Acceptability 2. Feasibility 3. Fidelity 4. Effectiveness 5. Short-term Sustainability	- Participants' feedback about the program using the Working Alliance Inventory (WAI) for patients - Consistent attendance of adolescents to the program as recorded in the attendance register - Facilitators' (health care providers') feedback using the Working Alliance Inventory for facilitators - Improved clinical and mental health outcomes using baseline and post intervention results of viral load and psychological assessments (Adherence, Stigma, Strengths and Difficulties, Stigma, Self Esteem, Quality of Life, Working Alliance, Anxiety, Depression, Clinical Outcomes in Routine Evaluation—Outcome Measure) - Streamlining manual use in HIV programming at CCC

**Table 2:** Overview of theme and activities.

**Session 1: HIV/AIDS, Disclosure, Treatment, and adherence** Activity 1: Bead making Activity 2: Knowledge about HIV Activity 3: Disclosure Activity 4: Treatment buddy Activity 5: Adherence Activity 6: Wrap-up **Session 2: Adolescent development, Relationships, Sexual and reproductive health** Activity 1: Recap Activity 2: Adolescent development Activity 3: Relationships Activity 4: Sexual rights Activity 5: Contraceptives Activity 6: Wrap-up **Session 3: Feelings, Stigma and discrimination, Communication and Problem solving** Activity 1: Recap Activity 2: Feelings Activity 3: Stigma and discrimination Activity 4: Communication Activity 5: Problem solving Activity 6: Wrap-up **Session 4: Values, Decision making, Goal setting, Studies and the future** Activity 1: Recap Activity 2: Values Activity 3: From values to decision making using critical thinking Activity 4: Goal setting, Studies and the future Activity 5: Vision board Activity 6: Wrap-up **Session 5: Substance Abuse, Peer Pressure, Nutrition, Self-care and Positive living** Activity 1: Recap Activity 2: Social influences; drugs and alcohol, media Activity 3: Food and Nutrition—sandwich making Activity 4: Hygiene and self-care Activity 5: Body mapping Activity 6: Wrap-up **Session 6: Support systems and Transitioning** Activity 1: Recap Activity 2: Support systems—hand activity Activity 3: Social supports we can tap into Activity 4: Transitioning Activity 5: Wrap-up **Session 7: Assessments, way forward and prepare for termination** Activity 1: Assessments Activity 2: Recap Activity 3: Way forward after termination, plans, goals (individual and group) Activity 4: Wrap-up **Session 8: Termination** Activity 1: Recap and feedback on assessments Activity 2: Setting up of support system Activity 3: Certificates ceremony for completion of program

#### Participants

We will engage various stakeholders to give their experiences, interests, and expertise on young people and the psychosocial issues they face with a focus on YLHIV. Potential stakeholders include persons with expertise in HIV/AIDS and young people: we expect representation from governmental, non-governmental, and academic institutions, caregivers of YLHIV, and clinicians and peer-mentors who work with this group. We believe that engaging a variety of players will shape the contributions made, thereby enhancing our manual.

#### Data Collection Method

Data will be collected in the form of recorded interviews and notes. We will then use this information to develop three similar, yet age-differentiated manuals and workbooks. Table 2 highlights an overview of the themes and activities expected for each session. We describe in detail the process by which we will engage and collaborate with the stakeholders in Wambua et al. (36).

### Phase 2: The Pilot Trial

#### Study Design and Setting

The pilot trial will make used of a mixed-methods approach. The study will be conducted at the outpatient Comprehensive Care Center (CCC) at Kenyatta National Hospital (KNH), Kenya's largest national teaching and referral hospital in East and Central Africa. Records indicate that there are over 9,500 patients enrolled in care at the CCC, with about 10% representing young people aged between 10 and 24 years. The inclusion criteria for our pilot will include young people aged 10–24 years who are active in care at the CCC, know their status, have knowledge in both English and Kiswahili, will be available to participate in all eight sessions, and can give assent/consent and caregiver consent in case of minors. We acknowledge that some may have HIV-associated neurocognitive deficits and severe psychopathology (HIV-related psychosis, pervasive childhood disorders, severe depression, or suicidal ideation). During our baseline assessment, those identified as being impaired or with significant distress will be excluded from the trial as this may interfere with their ability to participate in the group activities.

#### Participant Timeline

Participants who provide assent/consent to participate in the study will be enrolled for a period of 24 months. The intervention program will take ~4 months from the baseline assessment with sessions held every fortnight. After completion of the intervention, participants receive a follow-up review and assessments after 6 and 12 months.

#### Sample Size

We will use a convenient sample of 28 participants from the already existing eligible patients attending the CCC at KNH aged between 10 and 24 years. In addition, we will use a proportional allocation formula to obtain the proportional size for each of the proposed groups: Tumaini−7, Amani−6, and Hodari−15. The sample for each group will be chosen based on the availability of the young people. Inclusion in the pilot will be based on availability to participate in all eight sessions, which will take place fortnightly on Saturday morning. Many of the young people between the ages of 10–18 years are usually in school—either boarding school or attending school on Saturdays. As such, they will be unable to attend the eight sessions. Therefore, we will recruit fewer participants from these two groups. Having the three groups ensures that activities in the workbooks and discussions address age-specific needs. For the proposed study, the sample size, though small, is reflective of the average number of participants in a typical support group at the CCC at KNH, which is 30, and will be representative of the target population at the CCC. Using this sample size, it will be possible to derive important information that is relevant for the use of this manual. As this is an exploratory trial, though it is underpowered, it is aimed at streamlining a developed intervention at the CCC. This information will supplement already existing data from previous support groups conducted at the CCC.

#### Data Collection Instruments and Procedure

Quantitative data will be collected using laboratory measures of Viral load and CD4 as well as assessment tools that include a researcher designed Socio-demographic questionnaire, socio-psychological tools such as the Patient Health Questionnaire−4 and 9 (PHQ-4 and PHQ-9) (37), Morisky Medication Adherence Scale, Internalized HIV related Stigma Scale, Rosenberg Self-Esteem Scale (RSES) (38), WHO Quality of Life Scale (WHO-QoL) (39), Strengths and Difficulties Questionnaire (SDQ) (40), Young Persons—Clinical Outcomes in Routine Evaluation (YP-CORE) (41), Working Alliance Inventory (WAI) (42), and the Patient Satisfaction Questionnaire and Facilitator Feedback Form. Qualitative data will be collected in the form of life stories, focus group discussions, and semi-structured interviews with the adolescent participants and peer-facilitators.

At baseline assessment, the participants will complete all the above-mentioned assessments (except the WAI) and information will also be gathered concerning their demographic background such as age, school or work, who they live with, knowledge of medication they take (as well as the time they take it and who reminds them), and disclosure (when did they find out, have they disclosed to others). Information on viral load and CD4 laboratory measures will be retrieved from the clinic's data system. After sessions, assessments will include the WAI, which will be filled by both the young participants and the peer-facilitators. Post-intervention follow-up assessments will include PHQ-9, YP-CORE, SDQ, and RSES and will be given immediately after the intervention ends (week 7) and at 6 and 12 month follow-ups. See Table 3 for a detailed description of how we will collect the quantitative data.

**Table 3:** Assessment schedule.

	**Baseline**	**After each session**	**End of intervention**	**Follow up: 6 and 12 months**
***Tumaini***				
Demographic questionnaire	X		X	X
Viral load	X		X	X
Medication Adherence	X		X	
Internalized Stigma	X		X	
Self Esteem	X		X	X
SDQ	X		X	
YP-CORE	X		X	X
WAI		X		
Participant satisfaction questionnaire			X	
***Amani and Hodari***				
Demographic questionnaire	X		X	X
Viral load	X		X	X
Medication Adherence	X		X	
Internalized Stigma	X		X	
Self Esteem	X		X	X
PHQ-4	X		X	
PHQ-9	X		X	X
WHO-QoL	X		X	
SDQ	X		X	X
YP-CORE	X		X	X
WAI		X		
Participant satisfaction questionnaire			X	
**Peer facilitators**				
WAI		X		
Facilitator feedback form		X		

#### Recruitment

**Figure 1:**
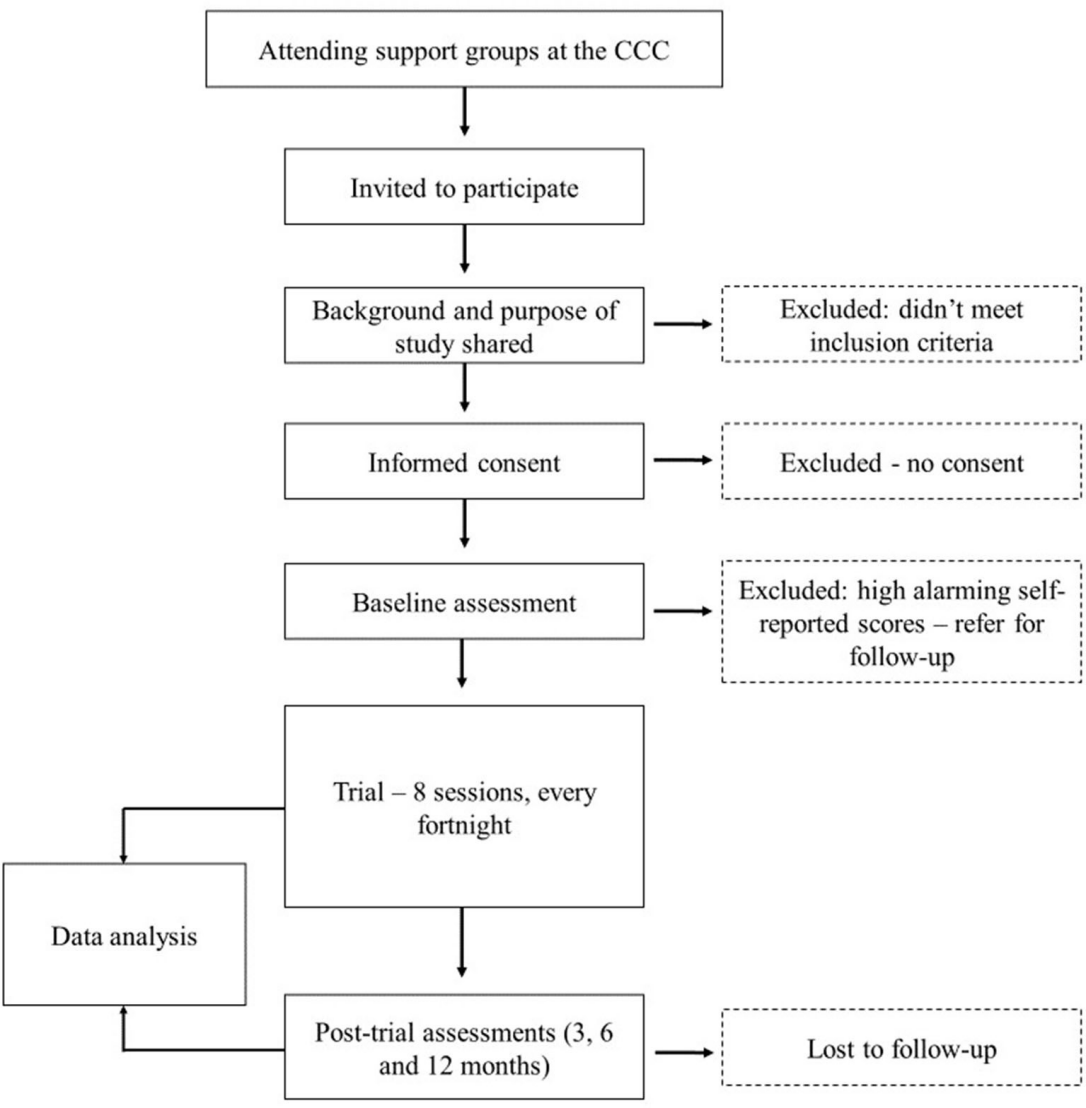
Flowchart of participant recruitment and follow-up.

Together with the help of the psychosocial team at the CCC, eligible participants will be targeted from the weekly support groups held on Wednesdays for 10–14 year-old, Thursdays for 15–19 year-old, and Fridays for 20–24 year-old. The young people and their caregivers will be briefed on the purpose of the study and will be given an opportunity to ask questions and seek clarification before they give formal assent/consent to participate in the study. See Figure 1 for a flow of participants.

#### Data Collection Method

The data collection procedure is described in Table 3. In this phase, data collection will be carried out through repeated assessments, focus group discussions, interviews, and audio-recording of the sessions on a fortnightly basis at baseline, end of the intervention, and 6 and 12 months after the intervention ends.

#### Intervention Strategy

During Phase 2 of the study, the impact of the intervention will be demonstrated by positive responses from participants and facilitators reflected in the four domains of acceptability, feasibility, fidelity, and short-term sustainability; this would fulfill the second aim of the study, which is to explore whether the manual content is usable by the target population.

#### Data Management

Data will be collected as described above and entered into a password protected database. Data will be extracted from the questionnaires using the double entry technique to minimize error. Questionnaires will be kept in a locked safe and soft copies will be password protected. Voice recordings of the sessions will be transferred into a password protected computer after every session, to be transcribed. The data collected will be destroyed after 5 years.

#### Data Analysis

The quantitative aspects of the data will be analyzed using statistical software, looking at changes in different factors as seen through the assessments at various stages of the program. We will conduct a descriptive analysis of the data using frequencies and percentages to describe the key features of the dataset. Continuous variables will be summarized using measures of central tendency and dispersion with a 95% confidence interval. We will then carry out bivariate analyses to examine the association between variables (Viral load, CD4, and other variables such as medication adherence, internalized stigma, self-esteem, depression, anxiety, quality of life, emotional and behavioral problems, and clinical outcomes). Changes in variables will be determined using the measures at baseline, endpoint, and at 6 and 12 months post intervention. These changes will be compared between the groups and within the groups and will be tested using independent and paired *t*-tests as appropriate. The focus group discussions and interviews will be translated where necessary and transcribed verbatim and coded into themes. Using content analysis, we will generate experiential categories from the data around the effectiveness of the program, peer facilitators' experience of providing support, how the young participants respond to the program, and its influence on their overall well-being.

#### Outcomes

Outcomes expected from the study include the development of the Positive and Healthy living manual and the conducting of an open trial using the manual to test its impact on the well-being of YLHIV. We will assess the impact outcomes on a multilevel scale:

- *Acceptability* of the manual and the activities will be assessed through the participants response and participation in the activities
- *Feasibility* will be seen through the consistent participation of the group held on a day outside their usual clinic day
- *Fidelity* will be seen through the peer-mentors improved skill and competencies related to knowledge, skills, and attitudes toward HIV/AIDS as they facilitate the sessions on a fortnightly basis
- *Short-term Sustainability* through the integration of the developed manual into psychosocial programming at the CCC will be assessed after the intervention.

### Ethics and Dissemination

We have obtained ethical clearance from the Kenyatta National Hospital/University of Nairobi Ethics Review Committee (P772/10/2016) and approval of the Head of CCC, KNH. Subsequent amendments to the protocol will be submitted to the ethical review board for approval.

The first and second author will meet with the potential participants and a thorough explanation of the study's purpose and process will be given to caregivers and young people prior to assenting/consenting. They will be informed that participation in the study is voluntary and that opting out at any time will not result in loss of services to the individual. Written informed assent/consent will be obtained from mature participants aged 18–24 years and emancipated minors aged 15–17 years, while written informed assent will be obtained from minor participants aged 10–17 years, in addition to the written informed consent obtained from their caregiver. For emancipated minors, a clinician overseeing their care will be requested to give consent on their behalf. Serial codes will be used on the questionnaires as identifiers instead of names. Questionnaires and audio-visual recordings will be stored in a locked safe. Data in soft copies are password protected and only accessible by the research personnel for the purpose of the study. Only the authors will have access to the data set. We do not expect any harmful physical effects to the participants. However, participants who may experience psychological distress during the intervention will be referred to our clinical team, comprising of psychiatrists and clinical psychologists, for further management. There will be no direct benefits for the participants. However, each participant will be given a compensation of Ksh 200 for transport on the days they will participate. In addition, they will be given snacks and tea after each group session for refreshment. On the last day of the trial, the participants will be offered lunch as some qualitative assessments might take a longer time. Indirectly, through their participation, YLHIV will be able to gain knowledge, skills, and a positive attitude to help them manage their health better. The findings of this study will also help other young people facing similar challenges to cope better. Participants' contribution to the study will help in the development of the Positive and Healthy Living Program manual, whose integration in support groups is expected to improve adolescent services at the CCC.

Our first point of dissemination will be to the KNH CCC Manager and Department of Mental Health In-charge, with a focus to exploring scaling up and discussing the short-term sustainability of group therapy for young people visiting the CCC. Our other dissemination plans include writing up the study for submission to peer-reviewed journals, highlighting the culturally relevant psychotherapeutic activities, as well as the training of peer counselors and findings of the trial. We will also invite our participants after the trial is over to share the findings of this work and invite their feedback on what could be done to boost psychosocial services to young people at the CCC. We will also share the study findings with the Ministry of Health Director of the Mental Health and Director of the National AIDS and STI Control Program, for their knowledge and comments about our intervention. The key outcome we expect from the dissemination is the incorporation of our program manual in the psychosocial programming at the CCC and possible scale up to other health facilities that offer HIV/AIDS support services.

### Study Limitations

Our study will be conducted on a small number of participants attending KNH CCC, which is in an urban area of Kenya. As such, the findings may not be generalized to young people living in rural settings. Further research that includes a larger number of participants from diverse geographical locations would yield more robust data that is generalizable. The study will not include participants who do not know their HIV status. Therefore, the findings may not be representative of all young people living with HIV in the age brackets included in the study. Studies that are more inclusive on HIV status of participants would uncover a wider impact of the intervention. Despite these limitations, we expect our study to identify multi-level facilitators and barriers to implementation of this unique home-grown intervention.

## Conclusion

Given the impact that psychosocial challenges have on the health and well-being of young people living with HIV, it is important to develop interventions that are culturally and age-appropriate to address these needs. Our findings will highlight the development of the “Positive and Healthy Living Program” intervention for support groups and its usability within health care settings. There has been no locally developed interventions for this age group and the program is expected to tap into specific psychosocial factors that contribute to poor adherence to treatment and high viral loads. Ultimately, it is hoped that the program will improve treatment adherence, confidence in negotiating difficult situations, emotions, and the general well-being of YLHIV. From a service delivery point of view, the manualized program will help the psychosocial teams provide nuanced care and allow them to upgrade their skills and integrate evidence-based practices in HIV programming. With KNH being the largest national referral and teaching hospital in East and Central Africa, it is imperative that the ongoing support groups for young people at the CCC be enhanced using the Positive and Healthy Living Manual in order to serve as a benchmark for other HIV care and treatment facilities in the country and the region. As such, through the collaborative effort of HIV treatment facilities, young people will be empowered through life transforming knowledge and life skills to reduce HIV/AIDS-related deaths.

## Data Availability Statement

The dataset used and/or analyzed during the current study are available from the corresponding author on reasonable request.

## Ethics Statement

Ethical approval was obtained from The Ethics and Research Committee of Kenyatta National Hospital and University of Nairobi (KNH-UON ERC ref no. P772/10/2016). All participants who were approached assented/consented to participate. Participants assented/consented to study findings being published in scientific journals and presented at conferences.

## Author Contributions

JM, GW, OM, and MK designed the study and wrote the paper. PM, DB, VO, RM, PN, and NO read, commented, and approved the manuscript. All authors read and approved the manuscript.

## Conflict of Interest

The authors declare that the research was conducted in the absence of any commercial or financial relationships that could be construed as a potential conflict of interest.

## Abbreviations

KNH: Kenyatta National Hospital
CCC: Comprehensive Care Center
HIV/AIDS: Human Immunodeficiency Virus/Acquired Immune Deficiency Syndrome
YLHIV: Young people living with HIV
ART: Antiretroviral Treatment
WHO-AA-HA, World Health Organization: Global Accelerated Action for the Health of Adolescents
UNAIDS: United Nations Programme on HIV/AIDS
SSA: Sub-Saharan Africa
NACC: National AIDS Control Council
APOC: Adolescent Package of Care
CETA: Common Elements Therapeutic Approach
NASCOP: National AIDS &
STI Control Programme
PHQ-4: Patient Health Questionnaire−4
PHQ-9: Patient Health Questionnaire−9
RSES: Rosenberg Self-Esteem Scale
WHO-QoL: WHO Quality of Life Scale
SDQ: Strengths and Difficulties Questionnaire
WAI: Working Alliance Inventory WAI
YP-CORE: Young Persons—Clinical Outcomes in Routine Evaluation.
